# Chloroplast Genomes Characterization of *Aconitum violaceum*, *Caltha palustris*, and *Delphinium denudatum* (Ranunculaceae)

**DOI:** 10.1002/ece3.72276

**Published:** 2025-10-07

**Authors:** Hui Li, Jingjing Jia, Abdullah, Abdul Sammad, Sayed Afzal Shah, Yuhua Huang, Ying Cui, Parviz Heidari, Xiaoxuan Tian

**Affiliations:** ^1^ State Key Laboratory of Chinese Medicine Modernization Tianjin University of Traditional Chinese Medicine Tianjin China; ^2^ Haihe Laboratory of Modern Chinese Medicine Tianjin China; ^3^ Department of Biological Sciences National University of Medical Sciences Rawalpindi Pakistan; ^4^ Faculty of Agriculture Shahrood University of Technology Shahrood Iran

**Keywords:** *Aconitum*, *Caltha*, chloroplast genome, *Delphinium*, phylogeny, Ranunculaceae

## Abstract

The Ranunculaceae family, encompassing approximately 2500 species across 50 genera, includes several taxa of medicinal importance. This study presents de novo assembled chloroplast (cp) genomes for three species: *Aconitum violaceum* Jacquem. ex Stapf, *Caltha palustris* L., and *Delphinium denudatum* Wall. ex Hook.f. & Thomson. All three cp genomes exhibited the typical angiosperm quadripartite structure—comprising a large single‐copy region, a small single‐copy region, and a pair of inverted repeats (IRa and IRb)—with total lengths of 154,523 bp ( *A. violaceum* ), 155,057 bp ( *C. palustris* ), and 154,228 bp ( *D. denudatum* ). Genome annotation identified 111–112 unique genes, including 77–78 protein‐coding genes, 30 tRNAs, and four rRNAs. Notably, *rps16* and *rpl32* were absent in *A. violaceum* and *D. denudatum* , whereas *infA* was missing in *C. palustris* . Comparative analysis revealed high synteny, with no major genomic rearrangements, although minor IR boundary shifts were observed involving *rps19*, *ycf1*, and *ndhF*. Codon usage showed a pronounced bias toward A/T‐ending codons (RSCU > 1.0), with leucine and isoleucine being the most frequently encoded amino acids. Simple sequence repeat (SSR) analysis detected 65–93 SSRs per genome, predominantly A/T‐rich mononucleotide motifs. Maximum likelihood phylogenetic analysis of 76 complete cp genomes confirmed the monophyly of *Aconitum* L., *Caltha* L., and *Delphinium* Tourn. ex L., placing *D. denudatum* within *Delphinium* and grouping *A. violaceum* with *Aconitum tanguticum* in subgenus *Aconitum*. These findings provide genomic resources to support improved phylogenetic resolution, molecular evolution studies, conservation genetics, and medicinal plant research within Ranunculaceae.

## Graphical abstract

The complete chloroplast genome sequences of *A. violaceum* , *C. palustris* , and *D. denudatum* have been sequenced in current study. We also performed detailed comparative chloroplast genomics of these species and also reconstructed their phylogeny.

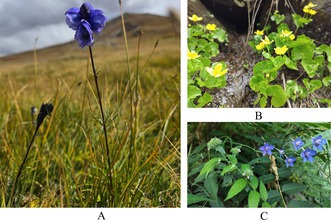

## 1 Introduction

The Ranunculaceae family comprises approximately 2500 species distributed across 50 accepted genera. These species predominantly inhabit temperate and subtropical regions, though some occur at high elevations within tropical zones (POWO 2025; Wang et al. 2009). Members of this family exhibit considerable morphological and ecological diversity, making them a focal point for studies in taxonomy, evolution, and medicinal plant research.

Among the most taxonomically rich genera within Ranunculaceae is *Aconitum* L., comprising around 343 accepted species (POWO 2025). These are primarily distributed across alpine and temperate regions of the Northern Hemisphere, particularly in mountainous areas such as the Himalayas and the Hengduan range, with southwestern China representing a notable center of diversity (POWO 2025; Kakkar et al. 2023). Eleven species and seven varieties of *Aconitum* are recognized in Pakistan (Riedl 1993). However, habitat degradation and unsustainable harvesting threaten several species worldwide, leading to their classification as endangered or vulnerable by the IUCN. One such species, *Aconitum violaceum* Jacquem. ex Stapf, inhabits Himalayan alpine slopes at elevations of 3500–4000 m, ranging across Pakistan, India, and Nepal (Kakkar et al. 2023). Valued in traditional medicine for its analgesic and anti‐inflammatory properties, these effects are attributed to bioactive compounds such as diterpenoid alkaloids (e.g., aconitine and indaconitine) and flavonoid glycosides (Ali et al. 2021; Safdar and Bibi 2020). These applications contribute significantly to its vulnerability through overexploitation.

Another genus is *Caltha* L., comprising approximately 16 species with a disjunct global distribution. *Caltha palustris* L. (marsh‐marigold or kingcup) is among the most widespread, occurring throughout Europe, North America, and Asia, including Pakistan, India, and China (POWO 2025). Fresh tissues contain protoanemonin, a toxin that can cause severe gastrointestinal irritation and, in extreme cases, organ failure (Lee and Sung 2021). However, drying or thermal treatment converts protoanemonin into the less toxic anemonin, enabling the traditional use of processed *C. palustris* for its anti‐inflammatory, diuretic, and wound‐healing effects (Liakh et al. 2020).

*Delphinium* Tourn. ex L. represents another diverse genus, with approximately 537 species of annual and perennial herbs distributed mainly across the Northern Hemisphere and high‐altitude regions of tropical Africa (POWO 2025). *Delphinium denudatum* Wall. ex Hook.f. & Thomson (Jadwar), a perennial species native to the western Himalayas—including northeastern and eastern Afghanistan, Pakistan, Kashmir, and northwestern India—holds particular medicinal significance (Aleem et al. 2020; POWO 2025). Widely utilized in traditional Unani and Persian medicine as an antidote and treatment for neurological and cardiovascular ailments, its use dates back to the 10th century, as documented by Rhazes (Khory and Katrak 1903). Modern pharmacological studies corroborate its traditional uses, demonstrating antimicrobial, anxiolytic, anticonvulsant, analgesic, and hepatoprotective properties (Aleem et al. 2020).

Advances in high‐throughput sequencing have enabled the high‐quality assembly of nuclear, mitochondrial, and chloroplast (cp) genomes, facilitating research in evolutionary biology, supporting plant‐based drug discovery, and improving the accurate identification of species for the safe use of medicinal plants (Abdullah et al. 2025; Gao et al. 2023; Zhao et al. 2023). Chloroplast genomes are generally conserved in structure—featuring a quadripartite organization of large single‐copy (LSC), small single‐copy (SSC), and inverted repeat (IR) regions—and display uniparental inheritance and moderate levels of polymorphism. These features make cp genomes a valuable resource for research in plant systematics, population genetics, phylogenetics, conservation biology, and molecular barcoding (Ahmed et al. 2020; Daniell et al. 2016; Abdullah et al. 2025; Li, Zheng, et al. 2024; Xing et al. 2025; Zhang et al. 2023).

Several recent phylogenomic studies have investigated cp genomes within Ranunculaceae, providing insights into intrafamilial relationships and species evolution. These studies have generated valuable genomic resources for various genera, including *Aconitum*, *Caltha*, and *Delphinium* (Cheng and Xie 2014; Schuettpelz and Hoot 2004; Song et al. 2024; Xia et al. 2022; Zhai et al. 2019). However, the cp genomes of *A. violaceum* and *D. denudatum* have not yet been reported. Although *C. palustris* has been sequenced from Chinese populations, no cp genome data are currently available for the Pakistani population.

In this study, we report the complete cp genome sequences of *A. violaceum* , *C. palustris* (Pakistani accession), and *D. denudatum* . We characterized the genomes of these three species and reconstructed their phylogeny with related genera. This study may offer useful molecular resources for conservation genetics, medicinal plant research, and taxonomic revision.

## 2 Materials and Methods

### 2.1 Plant Collection, DNA Extraction, and Sequencing

**FIGURE 1:**
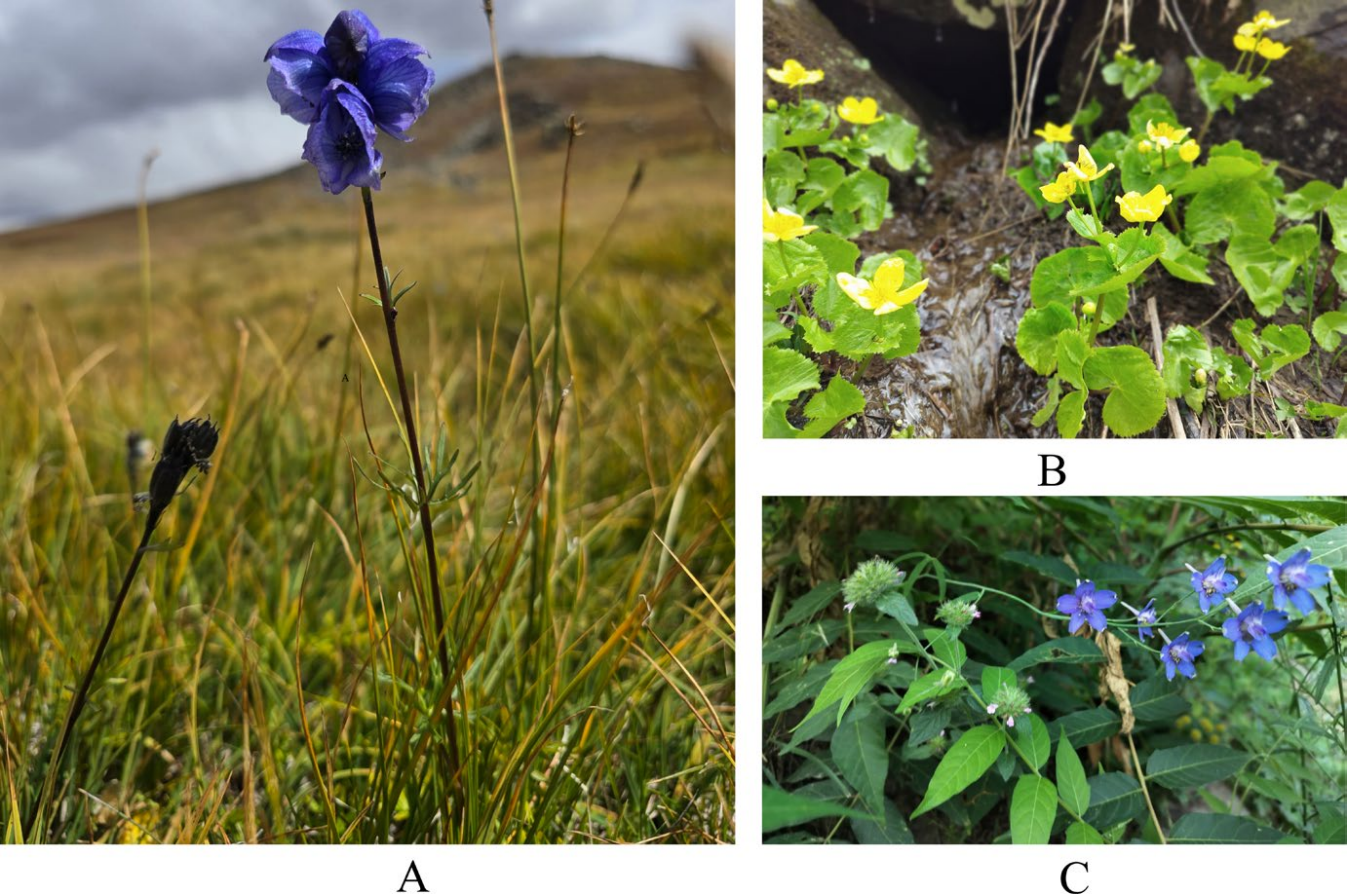
Representative photographs of species sequenced in the current study. (A) *Aconitum violaceum*, a plant with violet‐blue flowers borne on an erect stem. (B) *Caltha palustris* , and (C) *Delphinium denudatum*, with slender stems bearing racemes of violet‐blue flowers, surrounded by other species in its natural environment.

Plant material of *A. violaceum* was collected from Gilgit‐Baltistan, Pakistan (34°59′22″ N, 75°14′47″ E), whereas *C. palustris* and *D. denudatum* were collected from the Neelum Valley, Azad Kashmir, Pakistan ( *C. palustris* : 34°49′24″ N, 74°02′49″ E; *D. denudatum* : 34°47′31″ N, 74°11′22″ E) (Figure 1). No permission was required from the local authority or the central government of Pakistan for the collection of these plants or their parts, and for using them for research purposes in any way. Leaves of each sample were immediately desiccated in silica gel to preserve DNA integrity. Species identification was confirmed by Dr. Sayed Afzal Shah, and voucher specimens were preserved in the herbarium of the National University of Medical Sciences (Accession Nos.: NUMS00030 for *A. violaceum* , NUMS00034 for *C. palustris* , and NUMS00035 for *D. denudatum* ). Whole‐genome DNA extraction was performed using the Plant Genomic DNA Kit (TIANGEN BIOTECH, Beijing, China), following the manufacturer's protocol. Paired‐end sequencing (150 bp reads) was performed on the Illumina NovaSeq 6000 platform at Novogene (Tianjin, China).

### 2.2 Chloroplast Genomes Assembly and Annotation

Chloroplast genomes were assembled de novo using GetOrganelle v1.7.5.3 (Jin et al. 2020). Annotation was performed using GeSeq (Tillich et al. 2017), and the structural integrity of tRNA genes was validated with tRNAscan‐SE v2.0.7 (Chan and Lowe 2019). To ensure annotation accuracy, the assembled cp genomes were aligned with reference cp genomes of *Aconitum sinomontanum* Nakai (NC_036359), *C. palustris* (NC_041532), and *Delphinium grandiflorum* L. (NC_049872) using Geneious R8.1 (Kearse et al. 2012). Circular genome maps were generated with Chloroplot (Zheng et al. 2020) to visualize the structural features.

### 2.3 Genome Structure and Comparative Analysis

The assembled cp genomes were characterized in terms of genome size, gene content, GC composition, and lengths of the LSC, SSC, and IR regions using Geneious R8.1. Splicing patterns, including cis‐splicing and trans‐splicing genes, were identified and visualized with CPGview (Liu et al. 2023). Synteny and gene order conservation were evaluated using progressive Mauve alignment (Darling et al. 2004), and IR boundary regions were examined with CPJSdraw (Li, Guo, et al. 2023).

Relative synonymous codon usage (RSCU) values and amino acid frequencies were computed using custom Python scripts (Scripts 1 and 2 in Data S1 and S2). Simple sequence repeats (SSRs) were identified using MISA‐web online (https://webblast.ipk‐gatersleben.de/misa/) with the threshold settings as: 10 repeats for mononucleotides, 5 for dinucleotides, 4 for trinucleotides, and 3 for tetra‐, penta‐, and hexanucleotides (Beier et al. 2017).

### 2.4 Phylogenetic Reconstruction

Chloroplast genome sequences from 51 *Aconitum* species, 22 *Delphinium* species, and one *Caltha* species ( *C. palustris* ) were retrieved from NCBI GenBank and included in the phylogeny along with our sequenced species. *Asteropyrum cavaleriei* (H.Lév. & Vaniot) J.R.Drumm. & Hutch. (NC_041530) was included as an outgroup. Shared coding sequences (CDS) were extracted across all taxa and aligned using MAFFT v7 (Katoh and Standley 2013). The alignment was manually curated in Geneious R8.1 to remove ambiguous regions.

Maximum likelihood (ML) phylogenetic analysis was conducted using IQ‐TREE v3 (Minh et al. 2020). ModelFinder (Kalyaanamoorthy et al. 2017) identified the best‐fitting substitution model as TVM + F + R3. The final alignment of 76 sequences contained 68,949 nucleotide sites, including 61,142 constant sites (88.68%) and 4813 parsimony‐informative sites. Branch support was evaluated with 10,000 ultrafast bootstrap replicates and SH‐aLRT tests, incorporating the ‐bnni refinement option to reduce potential biases. Phylogenetic trees were visualized using the Interactive Tree of Life (iTOL) platform (Letunic and Bork 2019).

## 3 Results and Discussion

### 3.1 Chloroplast Genome Features, Gene Content, and GC Content Analysis

Whole‐genome sequencing generated substantial datasets for all three species. For *A. violaceum* , sequencing yielded 39.7 million raw reads (13.8 GB), with 0.45 million cp‐specific reads, supporting a high coverage of 438×. In *C. palustris* , 26.7 million reads (9.28 GB) produced a coverage depth of 265× from 0.27 million cp‐derived reads. *D. denudatum* had the highest coverage (571×), on the basis of 33.8 million reads (11.7 GB) and 0.59 million cp‐specific reads, which ensured high confidence in the cp genome assembly.

**FIGURE 2:**
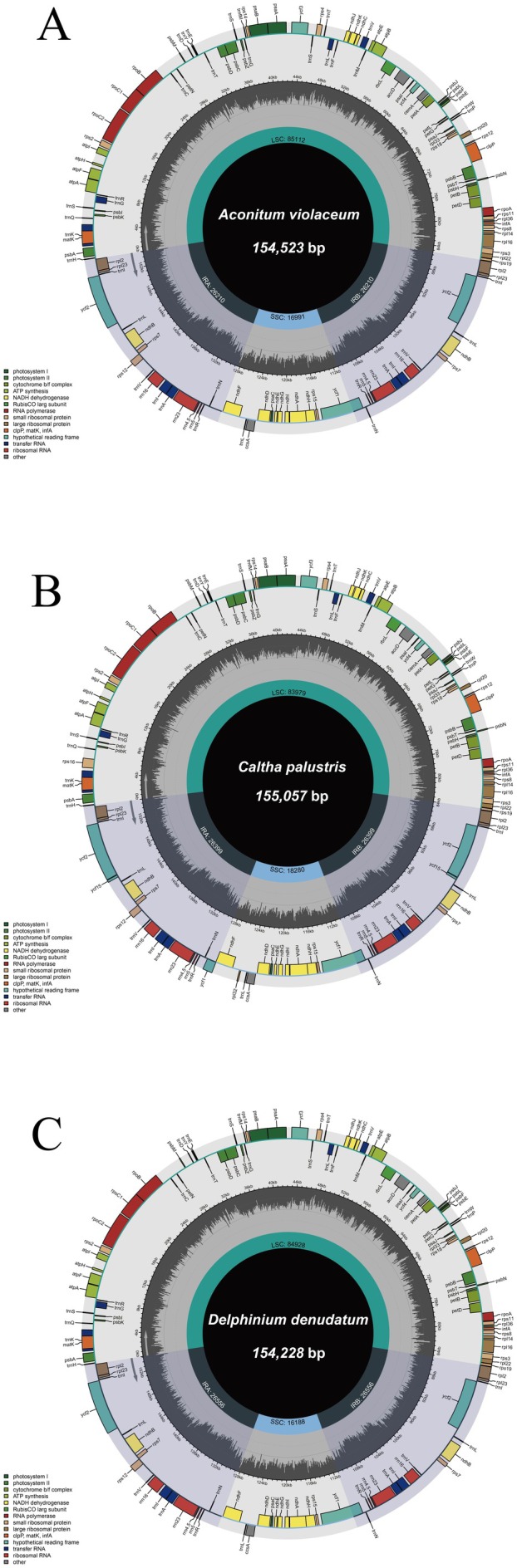
Circular chloroplast genome maps of (A) *A. violaceum* , (B) *C. palustris* , and (C) *D. denudatum* . The maps show the large single‐copy (LSC) region, small single‐copy (SSC) region, and two inverted repeats (IRa and IRb). Genes are color‐coded by functional category as indicated in the legend. Genes positioned on the outer circle are transcribed counterclockwise, whereas those on the inner circle are transcribed clockwise. The innermost gray histogram represents the variation in GC content across the genome.

All three cp genomes exhibited the conserved quadripartite structure, composed of an LSC, SSC, and IRs (IRa and IRb). The cp genome lengths were 154,523 bp in *A. violaceum* (LSC: 85,112 bp; SSC: 16,991 bp; each IR: 26,210 bp), 155,057 bp in *C. palustris* (LSC: 83,979 bp; SSC: 18,280 bp; each IR: 26,399 bp), and 154,228 bp in *D. denudatum* (LSC: 84,928 bp; SSC: 16,188 bp; each IR: 26,556 bp) (Figure 2), reflecting typical size variation within angiosperms.

**FIGURE 3:**
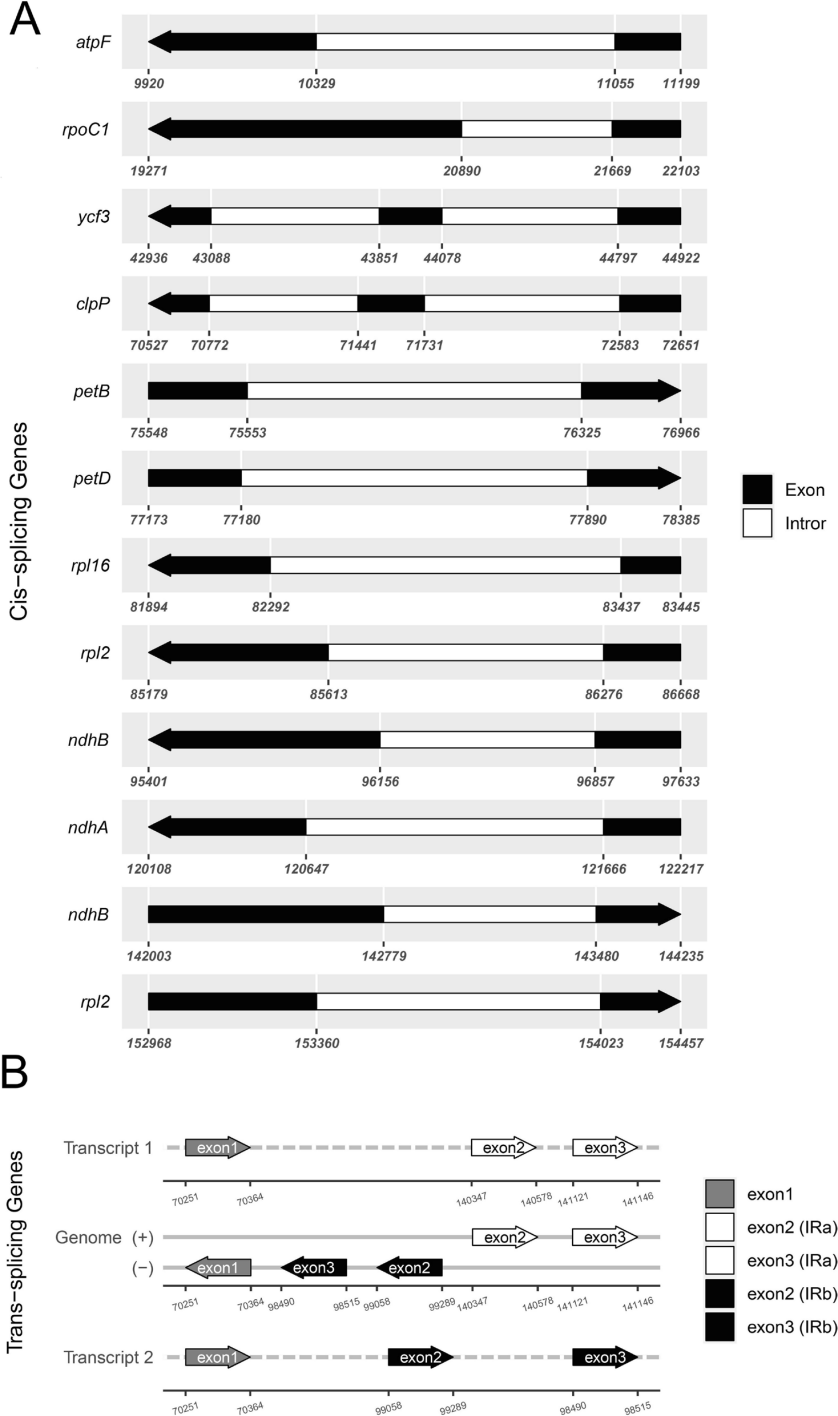
Schematic map of the cis‐splicing and trans‐splicing genes in the chloroplast genomes of *A. violaceum* . (A) The cis‐splicing genes of *A. violaceum* . (B) The trans‐splicing genes of *A. violaceum* .

Gene annotation revealed 111–112 unique genes, comprising 77–78 protein‐coding genes, 30 tRNA genes, and 4 rRNA genes. Both *A. violaceum* and *D. denudatum* lacked *rps16* and *rpl32*, whereas *C. palustris* showed an independent loss of *infA*. These gene losses or instances of pseudogenization are consistent with the variability observed in cp genome gene content across other genera (including *Aconitum* and *Delphinium*) within the Ranunculaceae family (Zhai et al. 2019). Similar patterns have also been observed in various other angiosperm lineages (Abdullah et al. 2020; Rehman et al. 2021; Song et al. 2024; Xia et al. 2022; Yanfei et al. 2023). Sixteen genes were duplicated in the IRs, including five protein‐coding genes (*ndhB*, *rpl2*, *rpl23*, *rps7*, *ycf2*), four rRNAs, and seven tRNAs (*trnA‐UGC*, *trnI‐CAU*, *trnI‐GAU*, *trnL‐CAA*, *trnN‐GUU*, *trnR‐ACG*, *trnV‐GAC*). In total, 16–17 intron‐containing genes were detected per species, with 10–11 undergoing cis‐splicing, and *rps12* was identified as a trans‐spliced gene (Figure 3, Figure S1; Table 1). These features reflect common patterns observed in monocot and eudicot cp genomes, including those of Ranunculaceae (Chen et al. 2024; Abdullah et al. 2025; Li, Abdullah, et al. 2025; Vo‐Tan et al. 2025; Zhai et al. 2019; Huang et al. 2025).

**TABLE 1:** Functional classification of genes in the chloroplast genome of *Aconitum violaceum*, *Caltha palustris* , and *Delphinium denudatum*.

Genes	Group of genes	Name of genes	Amount
Self‐replication	Large subunit of the ribosome	*rpl14*, *rpl16* ^a^, *rpl2* ^a^, ^b^, *rpl20*, *rpl22*, *rpl23* ^b^, *rpl33*, *rpl32* ^c^, *rpl36*	11
	Small subunit of the ribosome	*rps11*, *rps12* ^a^, *rps14*, *rps15*, *rps16* ^a^, ^c^, *rps18*, *rps19*, *rps2*, *rps3*, *rps4*, *rps7* ^b^, *rps8*	13
	DNA‐dependent RNA polymerase	*rpoA*, *rpoB*, *rpoC1* ^a^, *rpoC2*	4
	rRNA genes	*rrn16* ^b^, *rrn23* ^b^, *rrn4.5* ^b^, *rrn5* ^b^	8
	tRNA genes	*trnA‐UGC* ^a^, ^b^, *trnC‐GCA*, *trnD‐GUC*, *trnE‐UUC*, *trnF‐GAA*, *trnG‐GCC*, *trnG‐UCC*, *trnH‐GUG*, *trnI‐CAU* ^b^, *trnI‐GAU* ^a^, ^b^, *trnK‐UUU* ^a^, *trnL‐CAA* ^b^, *trnL‐UAA* ^a^, *trnL‐UAG*, *trnM‐CAU*, *trnN‐GUU* ^b^, *trnP‐UGG*, *trnQ‐UUG*, *trnR‐ACG* ^b^, *trnR‐UCU*, *trnS‐GCU*, *trnS‐GGA*, *trnS‐UGA*, *trnT‐GGU*, *trnT‐UGU*, *trnV‐GAC* ^b^, *trnV‐UAC* ^a^, *trnW‐CCA*, *trnY‐GUA*, *trnfM‐CAU*	37
Photosynthesis	Photosystem I	*psaA*, *psaB*, *psaC*, *psaI*, *psaJ*	5
	Photosystem II	*psbA*, *psbB*, *psbC*, *psbD*, *psbE*, *psbF*, *psbH*, *psbI*, *psbJ*, *psbK*, *psbL*, *psbM*, *psbN* (*pbf1*), *psbT*, *psbZ*	15
	NADPH dehydrogenase	*ndhA* ^a^, *ndhB* ^a^, ^b^, *ndhC*, *ndhD*, *ndhE*, *ndhF*, *ndhG*, *ndhH*, *ndhI*, *ndhJ*, *ndhK*	12
	Cytochrome b/f complex	*petA*, *petB* ^a^, *petD* ^a^, *petG*, *petL*, *petN*	6
	Subunits of ATP synthase	*atpA*, *atpB*, *atpE*, *atpF* ^a^, *atpH*, *atpI*	6
	Large subunit of Rubisco	*rbcL*	1
	Photosystem I assembly proteins^e^	*ycf3* ^a^ (*paf1*), *ycf4* (*paf1I*)	2
Other genes	Protease	*clpP* ^a^	1
	Maturase	*matK*	1
	Envelope membrane protein	*cemA*	1
	Subunit of Acetyl‐CoA‐carboxylase	*accD*	1
	C‐type cytochrome synthesis gene	*ccsA*	1
	Translation initiation factor	*infA* ^d^	1
	Conserved open reading frames	*ycf1*, *ycf2* ^b^	3
Total number of genes			129

Abbreviations: ATP, adenosine triphosphate; NADPH, nicotinamide adenine dinucleotide phosphate; rRNA, ribosomal RNA; tRNA, transfer RNA.

^a^ Introns containing genes.

^b^ Duplicated genes.

^c^ These genes were found missing in *Aconitum violaceum* and *Delphinium denudatum*.

^d^ The gene was found missing in *Caltha palustris* . The *pbf1*, *pafI*, and *pafII* were mentioned in brackets with *psbN*, *ycf3*, and *ycf4* as the GeSeq annotated them with these names.

^e^ The *ycf3* and *ycf4* are considered genes that belong to the category of conserved open reading frames. However, the functional studies also showed their role in the assembly of photosystem I (Boudreau et al. 1997; Naver et al. 2001; Nellaepalli et al. 2018).

**FIGURE 4:**
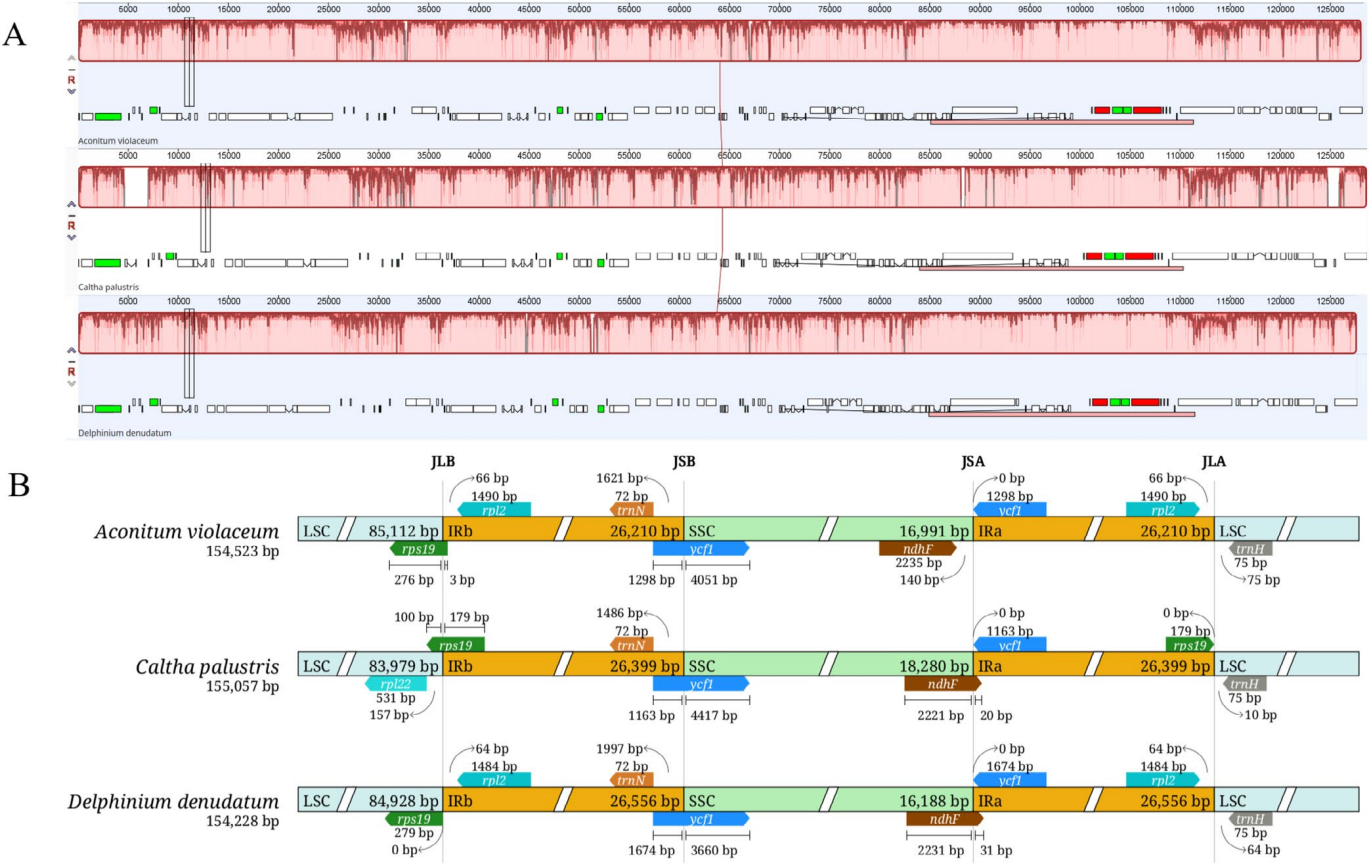
Comparative analysis of chloroplast genomes of *A. violaceum* , *C. palustris* , and *D. denudatum* . (A) Mauve alignment showing collinear blocks that indicate similarities in gene content and arrangement among the species. (B) Comparison of inverted repeat (IR) expansion and contraction. Junctions are labeled as follows: JLB = LSC/IRb, JSB = IRb/SSC, JSA = SSC/IRa, and JLA = IRa/LSC.

GC content was consistent across genomes and subregions among species (Table 2), aligning with previous reports (Song et al. 2024; Zhai et al. 2019). Mauve alignment also revealed a high degree of collinearity among the cp genomes of *A. violaceum* , *C. palustris* , and *D. denudatum* (Figure 4A), with no gene inversions or rearrangements detected and revealed a similarity in gene arrangements. The observed gene content, size range, and structural configuration suggest that these cp genomes retain the ancestral Type I architecture, defined by Zhai et al. (2019) as the most conserved among five structural types within Ranunculaceae. This conservation implies evolutionary stability and a lack of major genomic rearrangements since divergence from their last common ancestor, likely reflecting strong functional constraints on core photosynthetic machinery (Daniell et al. 2016; Zhang et al. 2023).

**TABLE 2:** Comparative analysis of the chloroplast genome of *Aconitum violaceum*, *Caltha palustris* , and *Delphinium denudatum*.

Characteristics		*Aconitum violaceum*	*Caltha palustris*	*Delphinium denudatum*
Size (base pair; bp)		154,523	155,057	154,228
LSC length (bp)		85,112	83,979	84,928
SSC length (bp)		16,991	18,280	16,188
IR length (bp)		26,210	26,399	26,556
Unique genes		111^a^	112^b^	111^a^
Protein‐coding genes		77	78	77
tRNA genes		30	30	30
rRNA genes		4	4	4
Duplicate genes		16	16	16
GC content (%)	Total	38.13	38.16	38.3
	LSC	36.25	36.45	36.37
	SSC	32.49	31.99	32.99
	IR	43.00	43.01	43.00
	CDS	38.35	38.33	38.51
	rRNA	55.54	55.43	55.51
	tRNA	52.89	53.26	52.72
	All gene	38.1	38.2	38.3

Abbreviations: bp, base pair; GC, Guanine‐cytosine; lR, inverted repeat; LSC, large single‐copy; rRNA, ribosomal RNA; SSC, small single‐copy; tRNA, transfer RNA.

^a^ The *rps16* and *rpl32* genes were found missing in these two species. Hence, the number of unique genes decreased.

^b^ The *infA* gene was found missing in *Caltha palustris* .

### 3.2 Inverted Repeats Contraction and Expansion

At the SSC/IRb junction (JSB), all species possessed the *ycf1* gene spanning the boundary, beginning in IRb (1163–1674 bp) and extending into the SSC region, where most of the sequence was located (3660–4417 bp). At the IRa/SSC boundary (JSA), all species contained a truncated *ycf1* ^*Ψ*^ pseudogene (1163–1674 bp), generated through partial duplication of the *ycf1* gene into the IR region (Figure 4B). The *ndhF* gene exhibited positional variability, being entirely confined to the SSC region in *A. violaceum* , but spanning the JSA boundary in both *C. palustris* and *D. denudatum* . In these species, 2221 and 2231 bp, respectively, were located in the SSC region, whereas 20 and 31 bp were incorporated into IRa. Both IRb and IRa harbored an intact *rpl2* gene, positioned 66 bp away in *A. violaceum* and 64 bp in *D. denudatum* from the LSC/IRb (JLB) and LSC/IRa (JLA) junctions. By contrast, because of IR contraction in *C. palustris* , *rpl2* was located in the LSC region, 157 bp away from the JLB junction. At the LSC/IRa junction (JLA), *C. palustris* contained an *rps19* ^*Ψ*^ pseudogene, resulting from the initiation of the *rps19* gene in IRa and its extension into the LSC region, with 179 bp located in IRa and 100 bp in LSC. This partial duplication led to the formation of a pseudogene at the JLA junction. The *trnH* gene was entirely confined to the LSC region, positioned 75 bp ( *A. violaceum* ), 10 bp ( *C. palustris* ), and 64 bp ( *D. denudatum* ) from the JLA (Figure 4B). These variations mirror patterns reported in other Ranunculaceae species and are likely attributable to minor IR expansions or contractions. The overall conservation of IR boundaries underscores the close relatedness of these three species and is consistent with previous findings in other members of the genera (Lee et al. 2018; Song et al. 2024; Xia et al. 2022; Yanfei et al. 2023).

### 3.3 Analysis of Relative Synonymous Codon Usage, Amino Acid Frequency, and Simple Sequence Repeats

**FIGURE 5:**
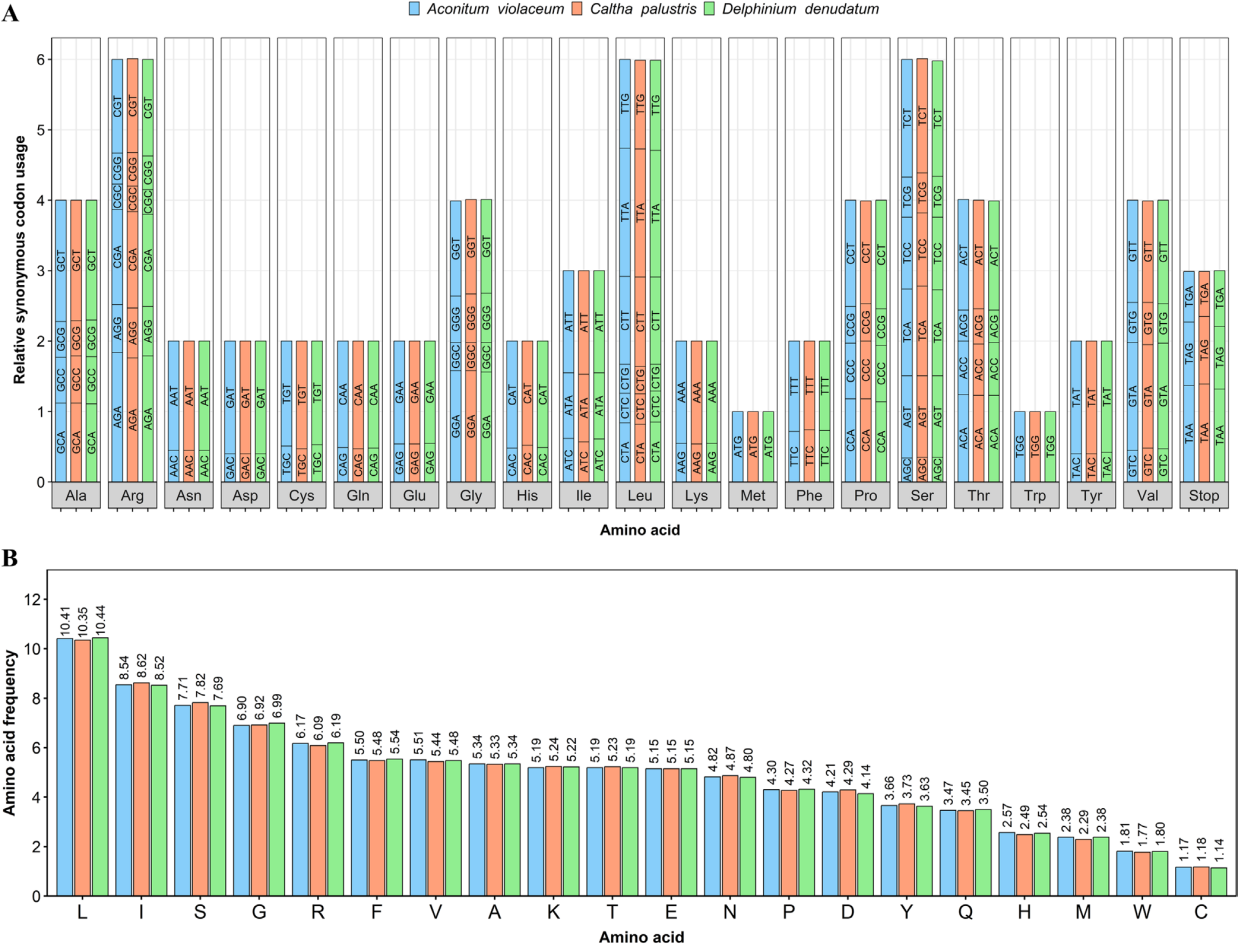
Codon usage bias and amino acid composition in the chloroplast genomes of *A. violaceum* , *C. palustris* , and *D. denudatum* . (A) Relative synonymous codon usage (RSCU). The *x*‐axis denotes amino acids, and the *y*‐axis shows the RSCU values for each species, with codons labeled within the bars. (B) Amino acid frequency distribution. The *x*‐axis represents amino acid types, and the *y*‐axis indicates their corresponding frequencies.

Codon usage analysis revealed a strong bias toward codons ending in A/T, with RSCU values > 1.0, whereas C/G‐ending codons were underrepresented (Figure 5A; Table S1). Leucine and isoleucine were the most frequently encoded amino acids, with cysteine being the least (Figure 5B; Table S2), consistent with reports of other angiosperms and also Ranunculaceae (Abdullah et al. 2021, 2025; Song et al. 2024).

**FIGURE 6:**
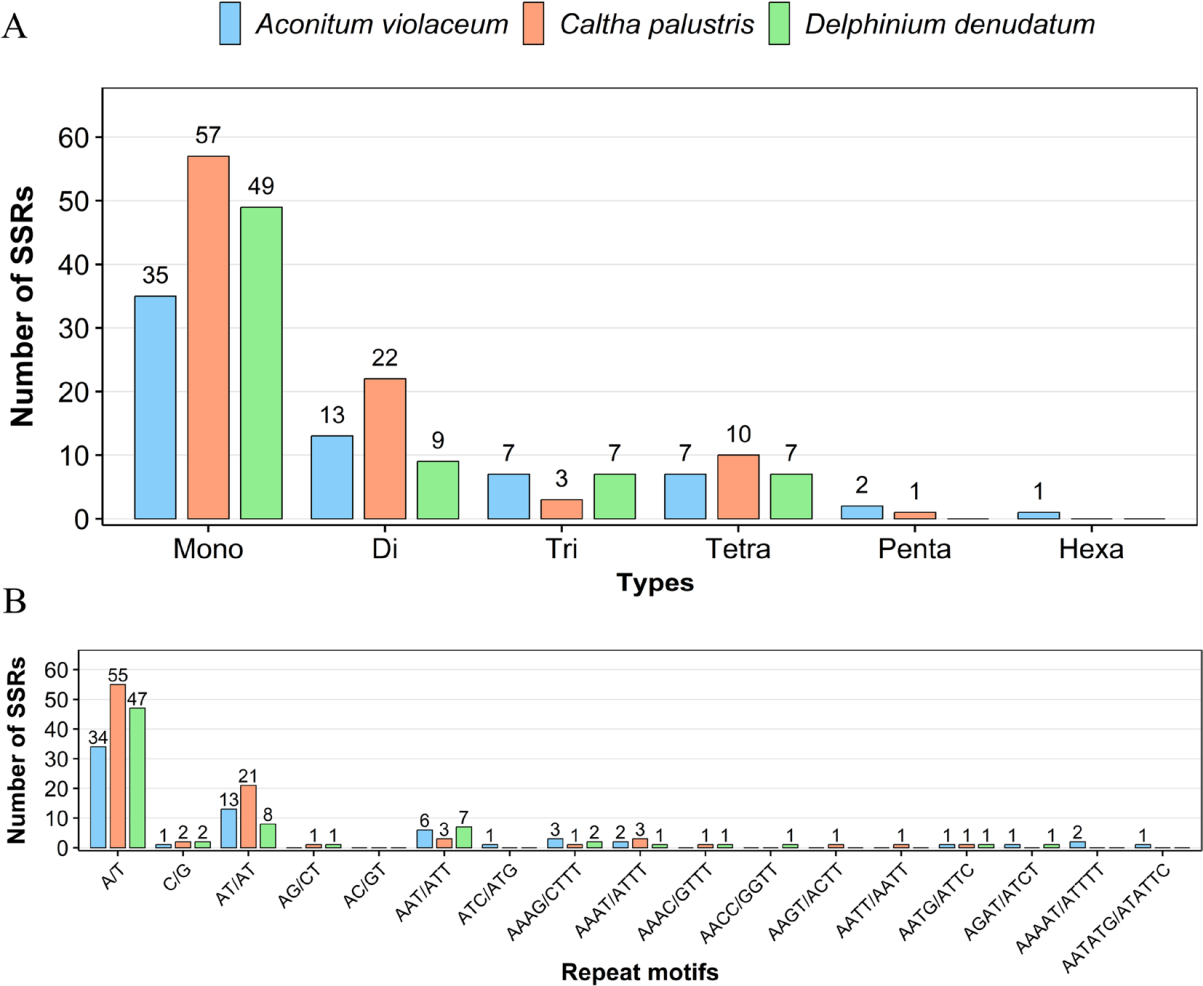
Comparison of simple sequence repeats (SSRs) in the chloroplast genomes of *A. violaceum* , *C. palustris* , and *D. denudatum* . (A) Distribution of SSR types. The *x*‐axis represents SSR types, and the *y*‐axis shows the number of SSRs. (B) Distribution of SSR motifs. The *x*‐axis denotes different SSR motifs, and the *y*‐axis indicates their corresponding counts.

Analysis of SSRs identified 65–93 SSRs per genome, primarily composed of A/T‐rich mononucleotide and dinucleotide repeats (Figure 6A,B; Tables S3 and S4). The abundance of A/T motifs reflects the general nucleotide bias of cp genomes and aligns with previous findings in the Ranunculaceae family (Li, Du, et al. 2023; Song et al. 2024; Huang et al. 2025). TheseSSRs may serve as useful markers for population genetics and phylogeographic studies, particularly valuable for assessing genetic diversity in threatened species like *A. violaceum* , which faces habitat fragmentation in the Himalayas.

### 3.4 Phylogenetic Analysis

**FIGURE 7:**
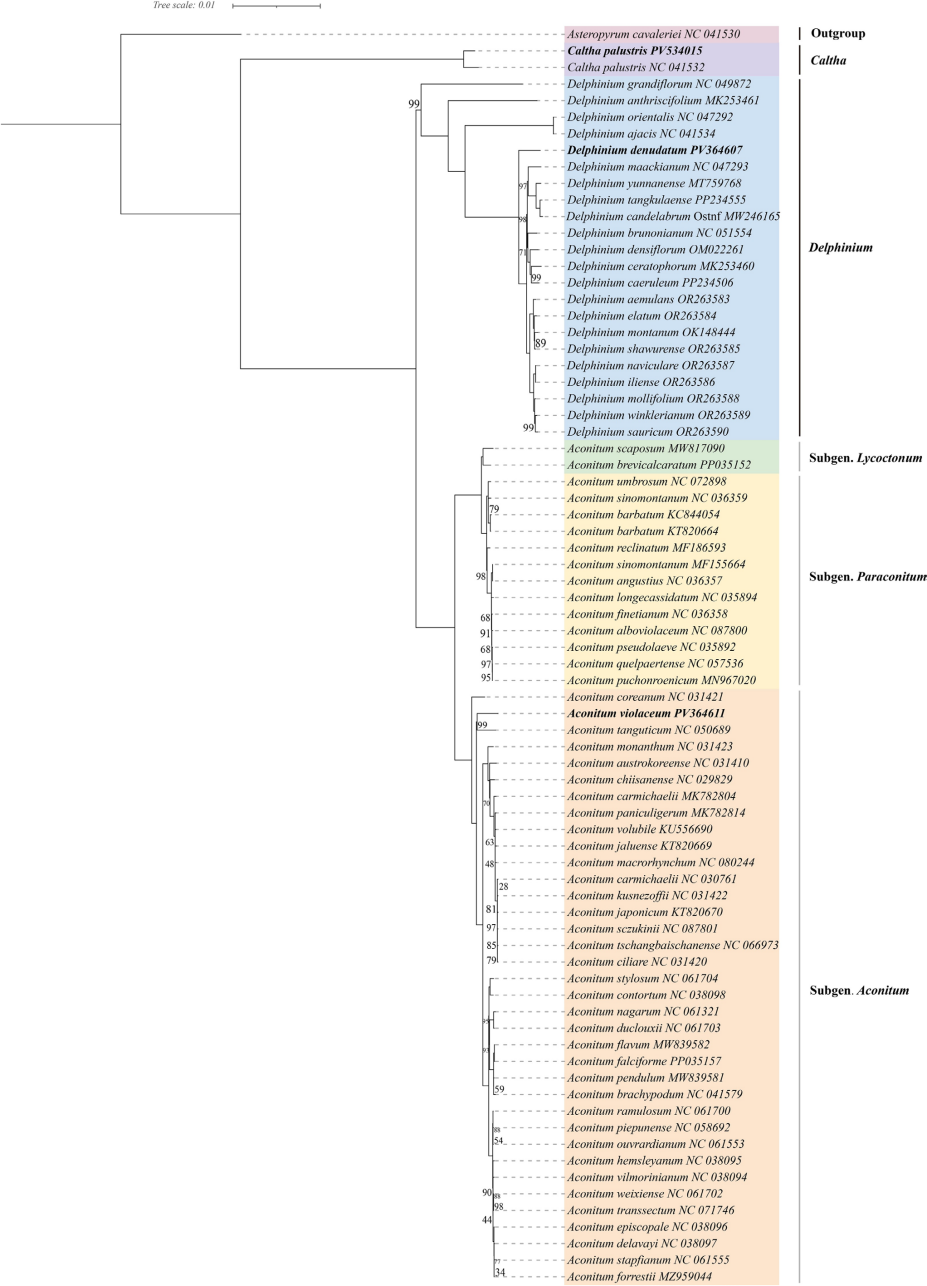
Phylogenetic tree of 75 Ranunculaceae species was reconstructed on the basis of 76 shared protein‐coding genes. The three newly sequenced species are highlighted in bold. *Asteropyrum cavaleriei* served as the outgroup. Numbers at each node indicate bootstrap support values. Branch lengths are proportional to genetic distance (scale: 0.01 substitutions per site). Bootstrap values equal to 100 are not shown for clarity.

Chloroplast‐based phylogenetic reconstruction clarified intergeneric and intrageneric relationships within Ranunculaceae (Figure 7). *Caltha palustris* (NC_041532, PV534015) formed a well‐supported sister lineage to the *Delphinium*–*Aconitum* clade (BS = 100%), consistent with its placement in tribe Caltheae, which diverges early from Delphinieae (Zhai et al. 2019). The robust monophyly of *Delphinium* (22 spp.) and *Aconitum* (51 spp.) supports the distinctiveness of these genera, despite reports of paraphyly in nuclear‐based phylogenies (Jabbour and Renner 2012). These discrepancies may arise from hybridization, cp capture, or incomplete lineage sorting—common in rapidly radiating lineages (Abdullah et al. 2021; Mehmood et al. 2020; Wen et al. 2018). Within *Delphinium*, the newly sequenced *D. denudatum* (PV364607) grouped within a robust subclade that includes two small clades containing many other *Delphinium* species such as *D. maackianum*, *D. yunnanense*, and *D. montanum* —all showing high bootstrap support. Within *Aconitum*, *A. violaceum* grouped with *A. tanguticum* (BS = 99%), and together they formed a clade sister to *A. coreanum* . This relationship places *A. violaceum* within Subg. *Aconitum*, the most derived clade following Subg. *Paraconitum* and basal Subg. *Lycoctonum*, aligning with prior taxonomic classifications (Jabbour and Renner 2012; Kong et al. 2017).

## 4 Conclusion

This study presents analyses of the chloroplast genomes of *A. violaceum* , *C. palustris* , and *D. denudatum* , revealing conserved genome structure, gene content, and IR boundaries. Codon usage analysis showed a preference for A/T‐ending codons, and amino acid frequencies were highest for leucine and isoleucine. SSR analysis identified 65–93 repeats, primarily mononucleotide A/T repeats, followed by dinucleotide repeats. Phylogenetic reconstruction resolved intergeneric and intrageneric relationships within Ranunculaceae with strong support. These results may provide valuable genomic resources and improve our understanding of chloroplast genome evolution, supporting future work in taxonomy, systematics, and conservation of these medicinally important taxa.

## Author Contributions

**Hui Li:** data curation (equal), formal analysis (equal), writing – original draft (equal). **Jingjing Jia:** data curation (equal), formal analysis (equal), writing – original draft (equal). **Abdullah:** conceptualization (equal), data curation (equal), formal analysis (equal), methodology (equal), software (equal), validation (equal), writing – original draft (equal), writing – review and editing (equal). **Abdul Sammad:** data curation (equal), formal analysis (equal). **Sayed Afzal Shah:** resources (equal), validation (equal). **Yuhua Huang:** formal analysis (equal), visualization (equal). **Ying Cui:** methodology (equal), writing – review and editing (equal). **Parviz Heidari:** conceptualization (equal), investigation (equal), methodology (equal), resources (equal), writing – review and editing (equal). **Xiaoxuan Tian:** conceptualization (equal), investigation (equal), methodology (equal), resources (equal), writing – review and editing (equal).

## Conflicts of Interest

The authors declare no conflicts of interest.

## Supporting information


**Figure S1:** ece372276‐sup‐0001‐FigureS1.jpg.

**Table S1:** ece372276‐sup‐0002‐TableS1.xlsx.

**Table S2:** ece372276‐sup‐0003‐TableS2.xlsx.

**Table S3:** ece372276‐sup‐0004‐TableS3.xlsx.

**Table S4:** ece372276‐sup‐0005‐TableS4.xlsx.

**Data S1:** ece372276‐sup‐0006‐DataS1.txt.

**Data S2:** ece372276‐sup‐0007‐DataS2.txt.

## Data Availability

The complete chloroplast genome sequences of *A. violaceum* , *C. palustris* , and *D. denudatum* have been deposited in NCBI GenBank under accession numbers PV364611, PV534015, and PV364607, respectively. The associated raw sequencing data and BioSample information are available under BioProject ID PRJNA1276170 (https://www.ncbi.nlm.nih.gov/search/all/?term=PRJNA1276170).
