# Supplementary figures and images for: Chloroplast Genomes Characterization of Aconitum violaceum, Caltha palustris, and Delphinium denudatum (Ranunculaceae)
Source: Ecol Evol. 2025 Oct 7;15(10):e72276. doi: 10.1002/ece3.72276 (PMC12501957; doi:10.1002/ece3.72276)

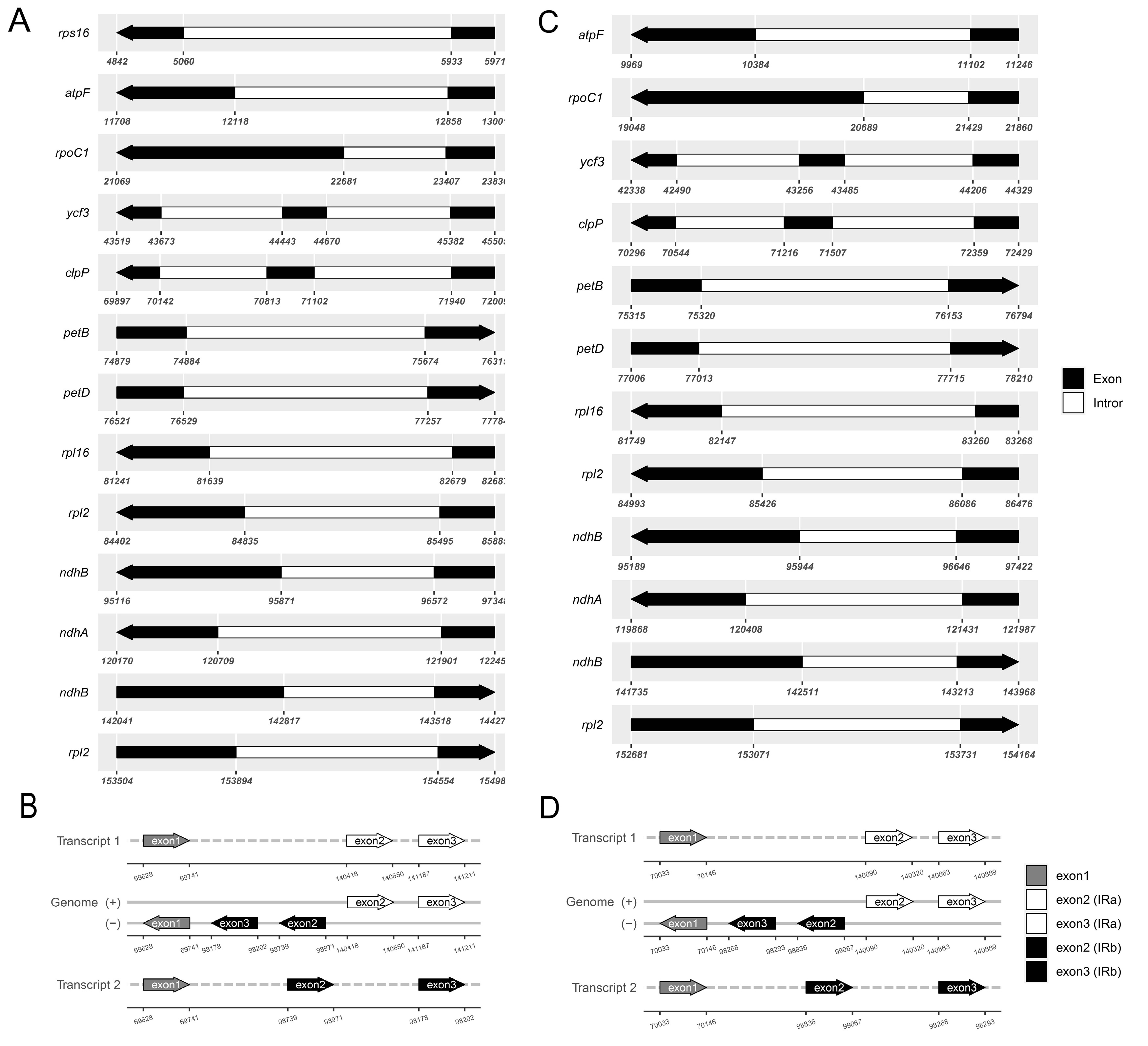

Supplement: Supplementary file 1 — Figure S1: ece372276‐sup‐0001‐FigureS1.jpg. [file ECE3-15-e72276-s004.jpg]
